# Points from Hospital Reports

**Published:** 1923-10

**Authors:** 


### POINTS FROM HOSPITAL REPORTS.

### Belgrave Hospital for Children.

The total ordinary expenditure of this hospital was a little less than in 1921, but an extra ward of ten beds was open from May, and there were 4,300 more attendances in the out-patients' department. The average cost per bed per week has been reduced to £3 3s. 5d., while the cost per out-patient attendance has dropped to 1s. 3d. A donation of £6,000 has been received, which enables the hospital to complete the erection of the South wing, which will contain the wards and eight nurse-sitting rooms and bedrooms.

### Paddington Green Children's Hospital.

A famous Frenchman once said: "Happy the people whose annals are blank in history's page." If that be applicable to the people who administer Hospitals, then none will gainsay us when we state that for one year at least the management of the Children's Hospital on Paddington Green have been in an enviable state of felicity. They have accomplished a full year's work; at the end of it their Account of Income and Expenditure exhibits what is cynically said to be the great desideratum of a Charity's accounts—a deficiency—and there have been no "events."

### Gloucestershire Royal Infirmary.

The salient features of this pithy and business-like Annual Report are a full year's work; progress marked by a number of valuable improvements; a decrease of ordinary expenditure; and the remarkable success of the Workpeople's Contributory Scheme, owing mainly to which the bank overdraft was reduced by £4,000. The report is made unusually interesting by comparative classified tabular statements of income and expenditure for a series of years, by means of which anyone can profitably compare one year with another, a most valuable but too rare feature of hospital reports; and by a graphic description in the Report of the General Committee, in illustration of the work done in the wards, of a very interesting case of ingenious operation on a child, by which its life was saved. This last is very effective yet judicious and dignified "window dressing." Small wonder that the Workpeople's Contributory Scheme is such a success, or that the churches in the district provide £1,000 a year. Such "touches of nature" move many whom the most skilfully prepared statistics leave cold.

### Royal Southern Hospital, Liverpool.

Commenting on the fact that only twelve service pensioners were numbered among the in-patients during 1922, the Committee observe that, "if the Ministry of Pensions continues to maintain its own hospitals, the services which the voluntary hospitals are called upon to render will soon become negligible." We are struck with the very small sum, only £161, received from workpeople's contributions. Compared with many other midland and north country hospitals, this is a very meagre figure, and we are not, therefore, surprised to read that "a serious endeavour is being made to bring about a closer co-operation between workpeople, employers and trade societies with a view to the establishment of a more general contributory scheme." The Committee refer appreciatively to the first appearance in the accounts of Grants from Approved Societies. They are, indeed, fitting, and should provide much more in the future. The total income of the year was sufficient not only to meet the expenditure, but to wipe out the deficiency of 1921.

### Glasgow Royal Infirmary.

This great hospital had under treatment in the wards last year 11,362 patients—541 more than in 1921. Indeed, the extent of the work in every department was greater than in the preceding year. The average length of stay of the in-patients was reduced from 23 to 21 days, and the rate of mortality among them was also less. Turning to finance, although the ordinary income fell considerably short of the ordinary expenditure, the extraordinary income more than made amends, so that there was an ultimate surplus of £15,000. There is in this report, in common with those of Scotch and English provincial hospitals generally, much more information of public interest than is to be found in most London hospital reports. Comparative tables of work and of income and expenditure, the numerical strength and composition of the hospital's complement, and other such statements add greatly to the value and interest of the report. We venture to suggest a minor but still useful improvement, the revision and alphabetical arrangement of the table of contents. Not everyone can find time to read the volume from cover to cover, and a complete alphabetical table of contents would much facilitate reference.

### Royal Hospital for Sick Children, Glasgow.

During 1922 a record amount of work was accomplished, the most notable fact, perhaps, in relation to it being that 5,089 children, drawn from all over Scotland, but chiefly from Glasgow and other parts of Lanarkshire, Dumbartonshire, Ayrshire and Renfrewshire, were in-patients. We notice with interest that the average stay of these patients in the hospital was 18 days, compared with 24 days in children's hospitals in London. Extensive organisations collect funds, respectively, from public works, warehouses, etc. (£3,500), and country districts (£2,100), and there is a Ladies' Auxiliary Association which collects £1,100.

